# The ALK inhibitor PF-06463922 is effective as a single agent in neuroblastoma driven by expression of ALK and MYCN

**DOI:** 10.1242/dmm.024448

**Published:** 2016-09-01

**Authors:** J. Guan, E. R. Tucker, H. Wan, D. Chand, L. S. Danielson, K. Ruuth, A. El Wakil, B. Witek, Y. Jamin, G. Umapathy, S. P. Robinson, T. W. Johnson, T. Smeal, T. Martinsson, L. Chesler, R. H. Palmer, B. Hallberg

**Affiliations:** 1 Department of Medical Biochemistry and Cell Biology, Institute of Biomedicine, Sahlgrenska Academy, University of Gothenburg, Gothenburg SE-405 30, Sweden; 2 Division of Clinical Studies Cancer Therapeutics, The Institute of Cancer Research, London and Royal Marsden NHS Foundation Trust, Sutton SM2 5NG, UK; 3 Department of Molecular Biology, Building 6L, Umeå University, Umeå 901 87, Sweden; 4 Division of Radiotherapy and Imaging, The Institute of Cancer Research, London and Royal Marsden NHS Foundation Trust, Sutton SM2 5NG, UK; 5 La Jolla Laboratories, Pfizer Worldwide Research and Development, San Diego, CA 92121, USA; 6 Department of Clinical Genetics, Institute of Biomedicine, Sahlgrenska Academy, University of Gothenburg, Gothenburg SE-405 30, Sweden

**Keywords:** PF-06463922, Neuroblastoma, ALK, MYCN, Mouse models, Lorlatinib

## Abstract

The first-in-class inhibitor of ALK, c-MET and ROS1, crizotinib (Xalkori), has shown remarkable clinical efficacy in treatment of ALK-positive non-small cell lung cancer. However, in neuroblastoma, activating mutations in the ALK kinase domain are typically refractory to crizotinib treatment, highlighting the need for more potent inhibitors. The next-generation ALK inhibitor PF-06463922 is predicted to exhibit increased affinity for ALK mutants prevalent in neuroblastoma. We examined PF-06463922 activity in ALK-driven neuroblastoma models *in vitro* and *in vivo*. *In vitro* kinase assays and cell-based experiments examining ALK mutations of increasing potency show that PF-06463922 is an effective inhibitor of ALK with greater activity towards ALK neuroblastoma mutants. In contrast to crizotinib, single agent administration of PF-06463922 caused dramatic tumor inhibition in both subcutaneous and orthotopic xenografts as well as a mouse model of high-risk neuroblastoma driven by *Th-ALK^F1174L^/MYCN*. Taken together, our results suggest PF-06463922 is a potent inhibitor of crizotinib-resistant ALK mutations, and highlights an important new treatment option for neuroblastoma patients.

## Abstract (precis)

**Summary:** Our results suggest that PF-06463922 is a potent inhibitor of crizotinib-resistant ALK mutations, and highlights an important new treatment option for neuroblastoma patients.

## INTRODUCTION

Anaplastic lymphoma kinase (ALK) is a clinical target of major interest in adult cancers characterized by chromosomal translocations (Hallberg and Palmer, 2013) in which the ALK kinase domain is fused to an array of amino-terminal partners, such as EML-4 (Rikova et al., 2007; Soda et al., 2007) in non-small cell lung cancer (NSCLC) (Awad and Shaw, 2014), or NPM (Morris et al., 1994) in anaplastic large cell lymphoma (ALCL). Crizotinib, the first and most completely characterized drug for treatment of these types of cancer, is an inhibitor of ALK, ROS1 and c-MET (Christensen et al., 2007) and exhibits robust activity in ALK fusion NSCLC patients, but treatment is complicated by the emergence of kinase domain point mutations and treatment resistance (Bosse and Maris, 2015; Hallberg and Palmer, 2013).

In recent years, a number of ALK inhibitors have been developed and are now approved for use in patients with NSCLC harboring ALK fusions (Awad and Shaw, 2014; Hallberg and Palmer, 2013). Second generation compounds with robust activity against both wild-type and activated resistance mutant ALK fusion proteins are in clinical trials. Ceritinib (LDK378) (Shaw and Engelman, 2014) was recently approved for use in crizotinib-relapsed ALK fusion-positive NSCLC in the US and Europe, and brigatinib (AP26113) (Katayama et al., 2011) has received a breakthrough therapy designation by the FDA (http://www.fda.gov/downloads/aboutfda/centersoffices/officeofmedicalproductsandtobacco/cder/ucm447165pdf, pp 11-15) (Table 1). Alectinib (CH5424802) has been approved in Japan for use in ALK fusion-positive NSCLC (Table 1). Other ALK inhibitors in trials include ASP3026 from Astrella (Katayama et al., 2012) (NCT01401504), and X-396 from Xcovery (Lovly et al., 2011) (NCT01625234) (Table 1). Although all are ATP-competitive inhibitors, these compounds differ in their binding properties, displaying differential activity in blocking the activity of the various ALK resistant mutant forms. Thus, a complex picture of ALK inhibition is emerging, with reports of distinct patterns of resistance mutations arising following primary treatment with particular ALK inhibitors (Friboulet et al., 2014; Katayama et al., 2014; Shaw and Engelman, 2014).

**Table 1.**
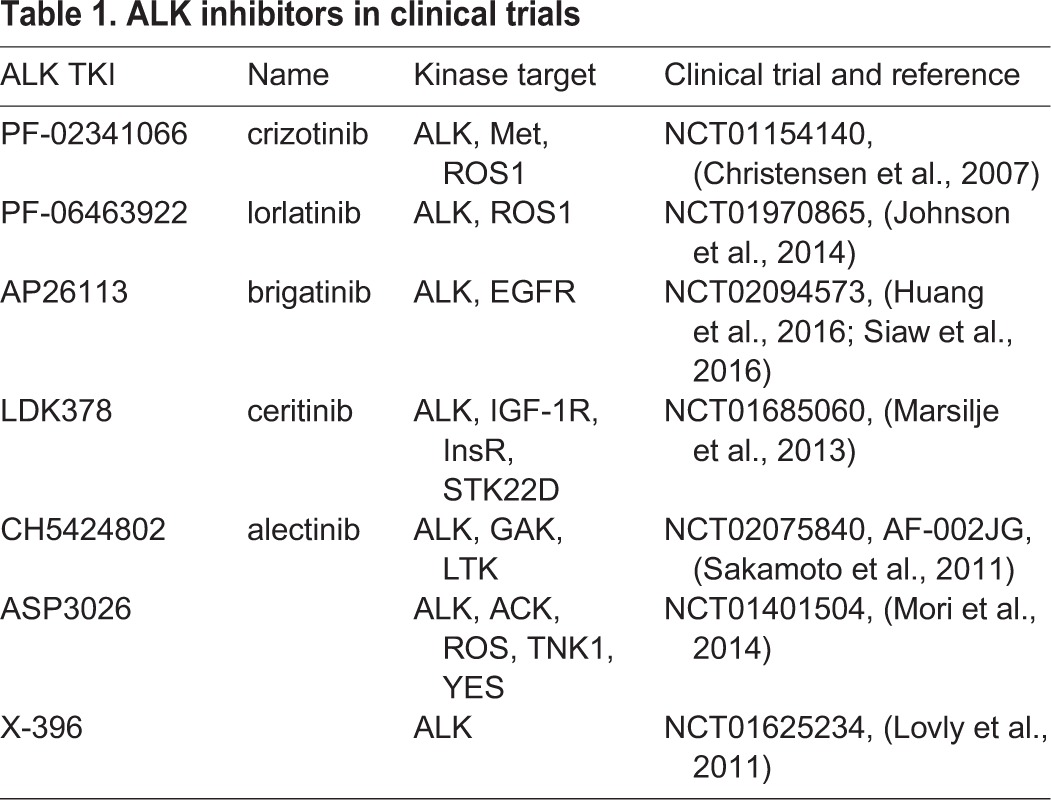
**ALK inhibitors in clinical trials**

The situation in pediatric malignancy is further complicated by point mutations that occur as primary and potentially driver mutations in therapy-naïve patients. Neuroblastoma, a childhood tumor of the developing nervous system, exhibits primary ALK mutations in ∼7% of samples, most commonly in the kinase domain, in both sporadic and familial forms (De Brouwer et al., 2010; Hallberg and Palmer, 2013; Schleiermacher et al., 2014). In addition, in neuroblastoma, subclonal ALK mutations can be present at diagnosis with subsequent clonal expansion at relapse (Eleveld et al., 2015; Schleiermacher et al., 2014). Recent identification of the ALK ligands FAM150A and FAM150B, which potently activate ALK signaling, raises the possibility that ALK activity may be important in non-ALK mutant neuroblastoma cases (Guan et al., 2015; Reshetnyak et al., 2015). A recent trial of crizotinib in ALK-positive pediatric cancers demonstrated excellent activity in ALCL, where NPM-ALK is the major driver. However, suboptimal activity was found in ALK mutant-positive neuroblastoma (Mosse et al., 2013). Mice in which the ALK^F1174L^ hotspot mutation and MYCN are co-expressed (*Th-ALK^F1174L^/MYCN*) develop neuroblastoma resistant to treatment with crizotinib (Berry et al., 2012). This model offers an ideal platform for the development of therapies that target expression of crizotinib-resistant ALK-positive neuroblastoma, such as combined ALK and mTOR inhibitors, or ALK and ERK5 inhibition (Berry et al., 2012; Umapathy et al., 2014).

Here we investigate the recently described ALK inhibitor PF-06463922 (lorlatinib) in neuroblastoma driven by expression of wild-type or mutant ALK, with a focus on whether PF-06463922 is more effective than crizotinib against those mutants known to confer crizotinib resistance. PF-06463922 is an orally available ATP-competitive selective inhibitor of ALK with excellent reported activity against both EML4-ALK as well as mutant EML4-ALK-resistant forms identified in patients treated with crizotinib (Huang et al., 2014; Johnson et al., 2014). The oral availability and pharmacokinetic-pharmacodynamics of PF-06463922 have been previously investigated in a preclinical lung cancer setting where anti-tumor activity was robust and independent of administration route (Yamazaki et al., 2014, 2015; Zou et al., 2015). Further, a preclinical evaluation of PF-06463922 displayed increased potency against acquired NSCLC ALK resistance mutations, with regression of EML4-ALK-driven brain metastases, and within the safety margins in a variety of preclinical studies (Johnson et al., 2014; Zou et al., 2015). Currently, PF-06463922 is in phase 1/2 clinical trials for treatment of ALK-driven cancer (https://clinicaltrials.gov/; NCT01970865). In this study we show that PF-06463922 is a highly effective inhibitor in neuroblastoma cell lines, as well as in non-native (Ba/F3 and PC12) cell model systems that express neuroblastoma-specific ALK mutations. The observed *in vitro* cellular sensitivity to PF-06463922 aligns with its ability to inhibit kinase activity. In addition, PF-06463922 is effective *in vivo* in both subcutaneous and orthotopic xenograft models of neuroblastoma, as well as the *Th-ALK^F1174L^/MYCN*-driven transgenic neuroblastoma mouse model. Taken together, our data suggest that PF-06463922 may be a superior treatment for ALK-positive neuroblastoma, defined by the presence of kinase-activating point mutations.

## RESULTS

### PF-06463922 abrogates growth in ALK-addicted neuroblastoma cell lines

**Fig. 1.**
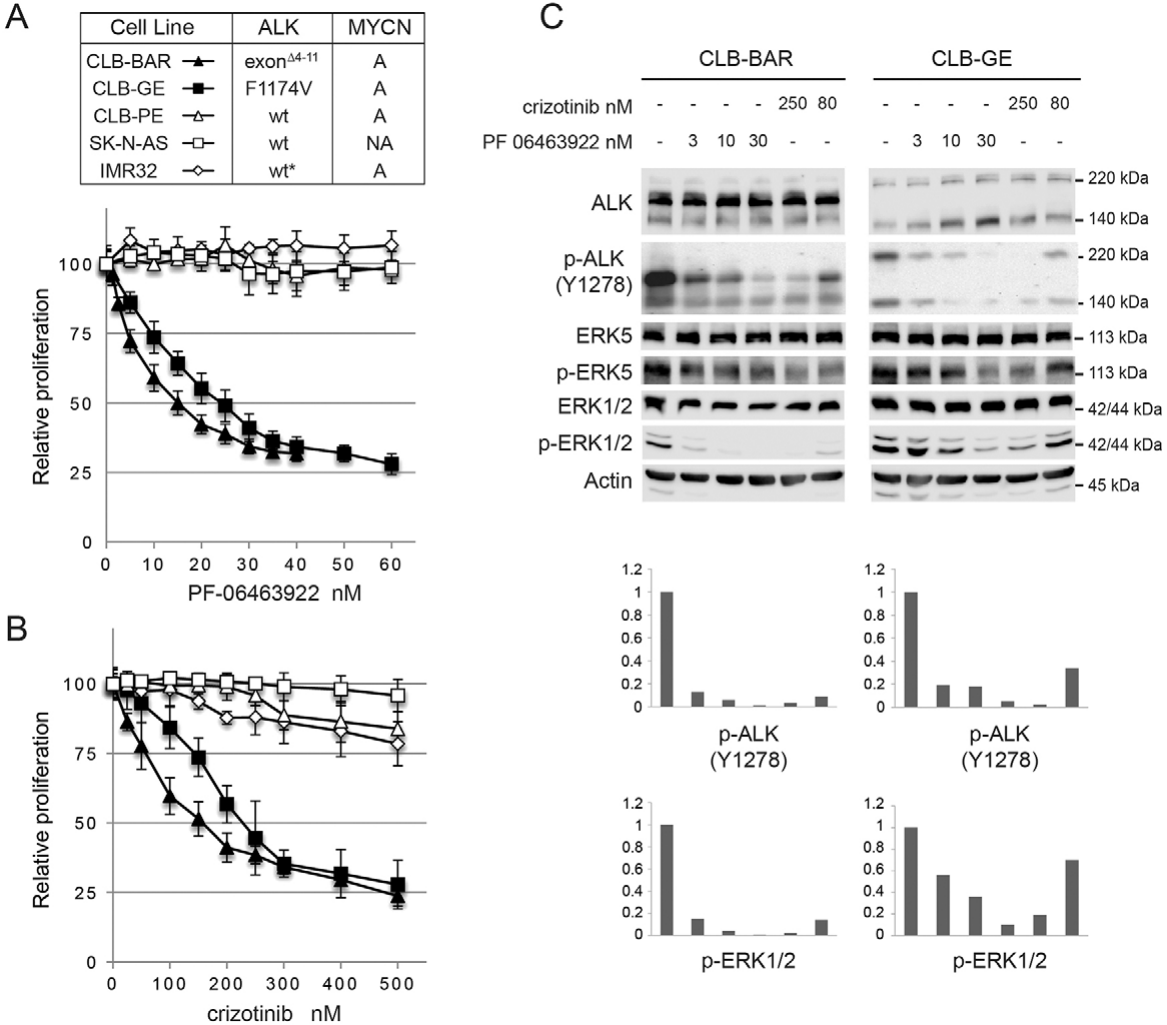
**Inhibition of neuroblastoma cell lines employing crizotinib and PF-06463922.** Treatment of neuroblastoma cells with the ALK TKIs crizotinib and PF-06463922. (A,B) Proliferation was assessed over 5 days using the resazurin cell proliferation assay in the following neuroblastoma cell lines: CLB-BAR, CLB-GE, CLB-PE, SK-N-AS and IMR32. Neuroblastoma cell line characteristics: CLB-GE – MYCN amplified, 1p deletion, 17q gain, amplified ALK amplicon containing an ALK^F1174V^ mutation; CLB-BAR – amplified MYCN/ALK, Δexon 4-11, 1p deletion, 17q gain; IMR32 – MYCN, wt sequence but exons 2-4 are amplified, 1p deletion, 17q gain; CLB-PE – 1p gain, amplified MYCN, 17q gain; SK-N-AS – IGF-1 overexpressing, 1p deletion, 1q gain, 17q gain, 17 deletion. Cells lines were treated with increasing concentrations of either PF-06463922 (A) or crizotinib (B) as indicated. Data are mean±s.d. of the fold-relative fluorescence from treated cells relative to untreated cells from three independent experiments. (C) CLB-BAR, CLB-GE neuroblastoma cells were treated for 6 h with either crizotinib or PF-04643922 as indicated. Cells were harvested and pre-cleared cell lysates were analyzed on SDS PAGE followed by western blotting for ALK, phospho-ALK-Y1278, phospho-ERK5, pan-ERK5 phospho-ERK1/2 and pan-ERK. Actin was employed as a loading control. Protein band intensities were quantified by Image Studio Lite 3.1 and normalized to untreated samples.

PF-06463922 is a recently developed small molecule tyrosine kinase inhibitor (TKI) that abrogates ALK activity (Johnson et al., 2014; Yamazaki et al., 2014). We examined the effect of PF-06463922 on ALK activity and cell proliferation in neuroblastoma cell lines, in comparison to the well-characterized clinical ALK TKI crizotinib (Christensen et al., 2007). We assembled a panel of neuroblastoma cell lines harboring mutated or wild-type ALK as follows: CLB-GE (amplified *MYCN/ALK, ALK^F1174V^*), CLB-BAR (amplified *MYCN/ALK*, *ALK Δexon 4-11*), IMR32 (*ALK exon 2-4* amplified *ALK*), CLB-PE (amplified *MYCN*) and SK-N-AS (non-amplified *MYCN*, WT *ALK*) (Cazes et al., 2013; Combaret et al., 1995; Fransson et al., 2015; Kohl et al., 1984; Schleiermacher et al., 2003). Cells were treated with increasing doses of either crizotinib or PF-06463922. Proliferation of both CLB-BAR and CLB-GE cell lines was inhibited in a dose-dependent manner by both crizotinib and PF-06463922 (Fig. 1A,B). Of the neuroblastoma cell lines tested, both CLB-BAR and CLB-GE are dependent on ALK activity and responded as ALK-addicted cell lines in a manner similar to that previously shown for crizotinib (Schonherr et al., 2012). Although both crizotinib and PF-06463922 inhibit growth, the IC_50_ values observed differ dramatically. For PF-06463922 IC_50_ values were 25±2 nM (mean±s.d.) in CLB-GE cells and 16±2 nM in CLB-BAR cells (Fig. 1B), whereas IC_50_ values for inhibition by crizotinib were 240±27 nM in CLB-GE and 157±37 nM in CLB-BAR cells (Fig. 1A). Importantly, we did not observe inhibition of proliferation in non-ALK-addicted cell lines such as CLB-PE, SK-NA-S and IMR32, which carry other driver mutations. Inhibition of proliferation by either PF-06463922 or crizotinib was accompanied by loss of tyrosine 1278 (Y1278) phosphorylation on ALK as well as inhibition of downstream signaling, e.g. pERK1/2 and pERK5 (Fig. 1C). We noted a slight decrease in proliferation of CLB-PE, SK-NA-S and IMR32 cells in response to high doses (500 nM) of crizotinib (Fig. 1B). This decrease in proliferation was not observed upon addition of high doses of PF-06463922 (Fig. 1A). In both cell lines increased levels of cleaved PARP were observed at 48 and 72 h of treatment indicating increased levels of apoptosis signal (Fig. S1A). Thus, PF-06463922 inhibits ALK activity in neuroblastoma cell lines dependent on ALK activity with high affinity and a lack of toxicity at the cellular level. Taken together, these preclinical results demonstrate that although crizotinib inhibits growth of the ALK-dependent neuroblastoma cells tested here, efficacy is increased on treatment with PF-06463922.

### PF-06463922 effectively inhibits constitutively active mutant ALK in Ba/F3 and PC12 cell model systems

**Fig. 2.**
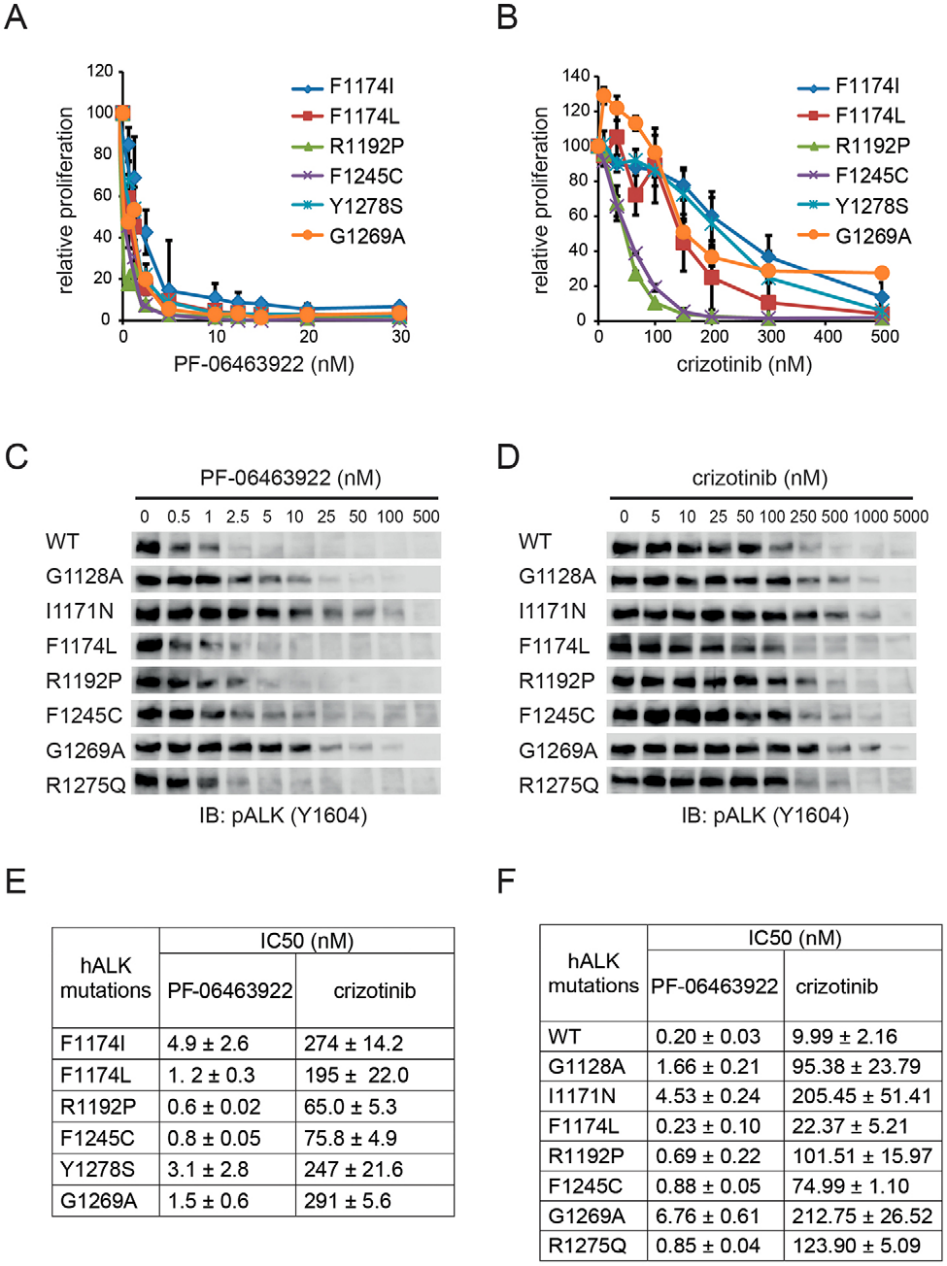
**Inhibition of ALK activity by crizotinib and PF-06463922 in Ba/F3 and PC12 cell systems.** (A,B) Ba/F3 cell analysis. IL-3-independent Ba/F3 cells expressing ALK^F1174I^, ALK^F1174L^, ALK^R1192P^, ALK^F1245C^, ALK^Y1278S^ or ALK^G1269A^ were treated with different concentrations of PF-06463922 (A) or crizotinib (B). Cell viability was assayed using resazurin after three days. Data are mean±s.d. of the fold-relative fluorescence from treated cells relative to untreated cells from three independent experiments. (C,D) PC12 cell analysis. Representative immunoblots of ALK autophosphorylation (phospho-ALK-Y1604) in PC12 cells overexpressing the indicated ALK mutant variants after treatment with increasing concentrations of PF-06463922 (0 to 500 nM) (C) or crizotinib (0 to 5000 nM) (D). (E,F) IC_50_ values for viability of Ba/F3 (E) and phospho-ALK-Y1604 in PC12 (F) cells overexpressing mutant ALK in response to treatment with crizotinib and PF-06463922. Values represent mean±s.d. from at least three independent experiments.

To extend our analysis, a murine interleukin-3-dependent pro-B cell line, Ba/F3, was employed (Chand et al., 2013; Lu et al., 2009; Schonherr et al., 2011). Ba/F3 cells are able to overcome IL-3 dependence, allowing them to survive and proliferate in the absence of IL-3 while expressing constitutively active tyrosine kinases or other oncogenes; characteristics that are often exploited in kinase drug discovery (Warmuth et al., 2007). In our previous analyses of mutant ALK reported from neuroblastoma patients, we have observed that not all of those tested that gave rise to foci in NIH3T3 transformation assays also exhibit Ba/F3 transforming potential. Although ALK^G1128A^, ALK^M1166R^ and ALK^R1275Q^ produce foci in NIH3T3 cells, they fail to confer IL-3-independent growth on Ba/F3 cells (Chand et al., 2013; Schonherr et al., 2011). In contrast, ALK^F1174I^, ALK^F1174L^, ALK^F1245C^, ALK^R1192P^ and ALK^Y1278S^ are all capable of producing foci in a NIH3T3 transformation assay and transforming Ba/F3 cells to IL-3-independent growth, and were thus analyzed here. Ba/F3 cells expressing mutant ALK receptors were treated with increasing doses of either crizotinib or PF-06463922 (Fig. 2A,B). ALK^F1174I^- and ALK^F1174L^-dependent IL-3-independent growth activity in the Ba/F3 system was blocked by crizotinib with relative IC_50_ values of 274 nM and 195 nM, respectively. ALK^F1174I^ and ALK^F1174L^ were inhibited by PF-06463922 with IC_50_ values of 4.9 nM and 1.2 nM, respectively, which are 50- to 160-fold lower as compared with crizotinib (Fig. 2E). All other ALK mutants tested were inhibited at IC_50_ values of between 65 to 300 nM crizotinib (Fig. 2E). IC_50_ values in response to PF-06463922 treatment ranged from 0.6 nM to 3.1 nM, which are 80- to 194-fold lower compared with crizotinib. We also examined the ALK^G1269A^ mutation, which is located −1 to the DFG of the kinase domain, as it is a secondary acquired resistance mutation described in NSCLC patients that is particularly resistant to inhibition by ALK inhibitors (Doebele et al., 2012). Although it has only been described in EML4-ALK fusions, we have included ALK^G1269A^ here in the context of full-length ALK, motivated by the potential future relevance for neuroblastoma patients that may be treated with ALK inhibitors. ALK^G1269A^ gives rise to foci formation in NIH3T3 transformation assays (data not shown) and IL-3-independent growth in Ba/F3 cells, and is effectively inhibited by PF-06463922 with an IC_50_ of 1.5 nM, compared with 291±5.6 nM for crizotinib (Fig. 2A,B,E).

In parallel we examined the activity of PF-06463922 in conjunction with gain-of-function ALK variants expressed in PC12 cells, which is a clonal rat adrenal pheochromocytoma cell line with enteric cell origin, with the ability to differentiate and extend neurites upon protracted ERK1/2 stimulation (Cowley et al., 1994). As a readout for ALK activity we employed phosphorylation of ALK Y1604, which reflects ALK activation (Chand et al., 2013). All loading ALK control blots for this experiment are shown as Fig. S2. We observed that wild-type ALK and ALK^F1174L^ were most sensitive to crizotinib inhibition of Y1604 phosphorylation, followed by ALK^F1245C^, ALK^G1128A^, ALK^R1192P^ and ALK^R1275Q^ (Fig. 2D,F). ALK^I1171N^ and the secondary resistant mutation mimic ALK^G1269A^ require a higher crizotinib dose for effective inhibition. A similar pattern of inhibition was observed with PF-06463922, although the amount of PF-06463922 required was substantially less (Fig. 2C,F). Similar to crizotinib, ALK^G1128A^, ALK^I1171N^ and ALK^G1269A^ demand higher doses of PF-06463922 to inhibit greater than 50% of the phosphorylation and activation of these ALK variants. In conclusion, as observed in the Ba/F3 analysis, PF-06463922 was 30- to 150-fold more potent as compared with crizotinib in inhibiting ALK phosphorylation and activation in PC12 cells. Similar patterns of inhibition were found for crizotinib and PF-06463922, with ALK^I1171N^ and ALK^G1269A^ in the full-length ALK receptor exhibiting increased resilience to inhibition when compared with the other gain-of-function ALK variants examined here.

### PF-06463922 effectively inhibits neuroblastoma ALK mutations in *in vitro* kinase assays

**Fig. 3.**
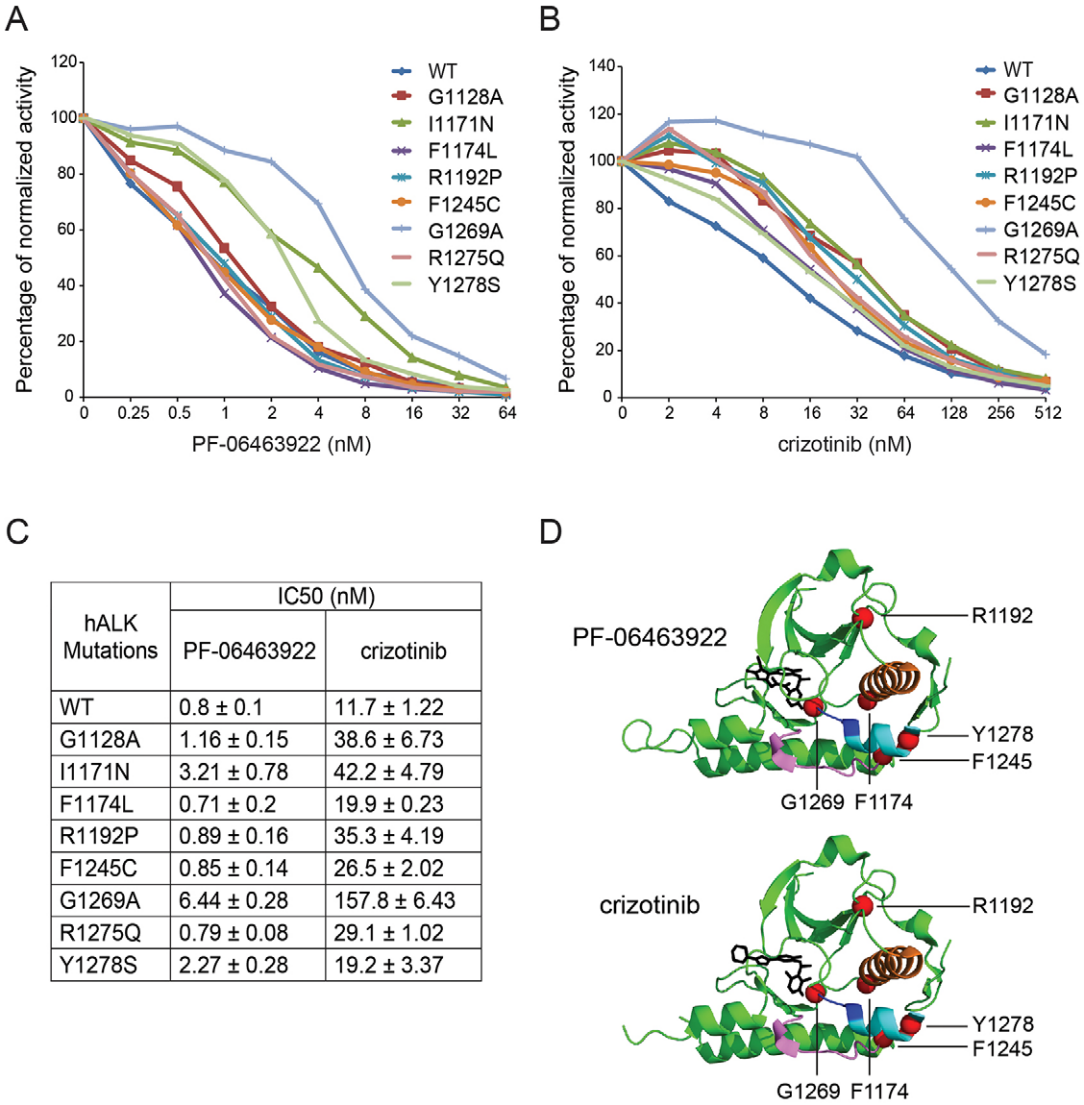
**Comparison of inhibition effects of crizotinib and PF-06463922 on WT and neuroblastoma gain-of-function mutant TKDs by *in vitro* kinase assay.** (A,B) Different ALK TKD proteins were incubated with either PF-06463922 (A) or crizotinib (B) in the presence of ATP (0.1 mM) and substrate peptides (0.2 mM). The incorporation of labelled γ-^32^P was detected under different conditions. Background counts from no-enzyme controls were subtracted, and the data were normalized to the 0 nM inhibitor reactions. (C) IC_50_ values from A,B were calculated by fitting data to a log (inhibitor) versus normalized response (variable slope) equation in GraphPad Prism 6.0. All data are shown as mean±s.d. from at least two independent experiments. (D) Crystal structures of ALK kinase domain in complex with PF-06463922 (top) or crizotinib (bottom). Compounds indicated in black. Gain-of-function ALK mutations F1174, R1192P, F1245, G1269 and Y1278 are shown as red spheres. The ribbon diagram displays αC helix (1157-1173; orange), catalytic loop (1246-125; magenta), activation loop (1271-1288; cyan) with DFG motif marked in blue. Figures were generated with PyMol using published coordinates (Protein data bank code: 4CLI and 2XP2).

Given the different responses of the various ALK mutants tested here in cell line backgrounds, we employed *in vitro* kinase assays to directly measure ALK activity. ALK kinase domains were produced and purified from baculovirus-infected *Spodoptera frugiperda* (Sf21) cells, which are a eukaryotic expression system with a high yield of recombinant protein expression (Ren et al., 1995). Direct measurements of the kinetics of inhibition by either crizotinib or PF-06463922 were performed (Fig. 3A,B). Only marginal differences in the kinetics of ALK kinase inhibition with crizotinib were observed between the ALK mutants *in vitro* (Fig. 3B,C). All mutants examined, with the exception of ALK^G1269A^, were inhibited by crizotinib at IC_50_ values of 1.5- to 3.5-fold of wild-type (Fig. 3B,C). In contrast, the ALK^G1269A^ kinase domain mutant is resistant to crizotinib inhibition with a 13.5-fold higher IC_50_ when compared with wild type (Fig. 3B,C). The ALK^G1269A^ kinase domain mutant was also the most resistant mutation when the response to PF-06463922 was investigated, with an IC_50_ eightfold that of wild type (Fig. 3A,C). The relative resistance of the ALK^G1269A^ kinase domain mutant derives from a close contact with this residue in the ATP/inhibitor binding pocket of ALK (Fig. 3D) (Huang et al., 2014; Johnson et al., 2014). Although ALK^G1128A^, ALK^F1174L^, ALK^R1192P^, ALK^F1245C^ and ALK^R1275Q^ responded to PF-06463922 with IC_50_ values similar to that of wild type, two mutants – ALK^I1171N^ and ALK^Y1278S^ – were more resistant, with 4- and 2.8-fold respectively of the IC_50_ of wild-type ALK kinase. Taken together, these *in vitro* kinase assay results suggest that although some ALK mutations are more sensitive than others, response to PF-06463922 is again improved over the response to crizotinib. In fact, all kinase domain mutations tested (Fig. 3C,D), including the most resistant secondary mutation – ALK^G1269A^ – respond to PF-06463922, with a 50% reduction in activity at inhibitor levels less than 6.5 nM, supporting PF-06463922 as a strong candidate for *in vivo* evaluation.

### PF-06463922 displays potent anti-tumor growth in both subcutaneous and orthotopic xenograft models of neuroblastoma

**Fig. 4.**
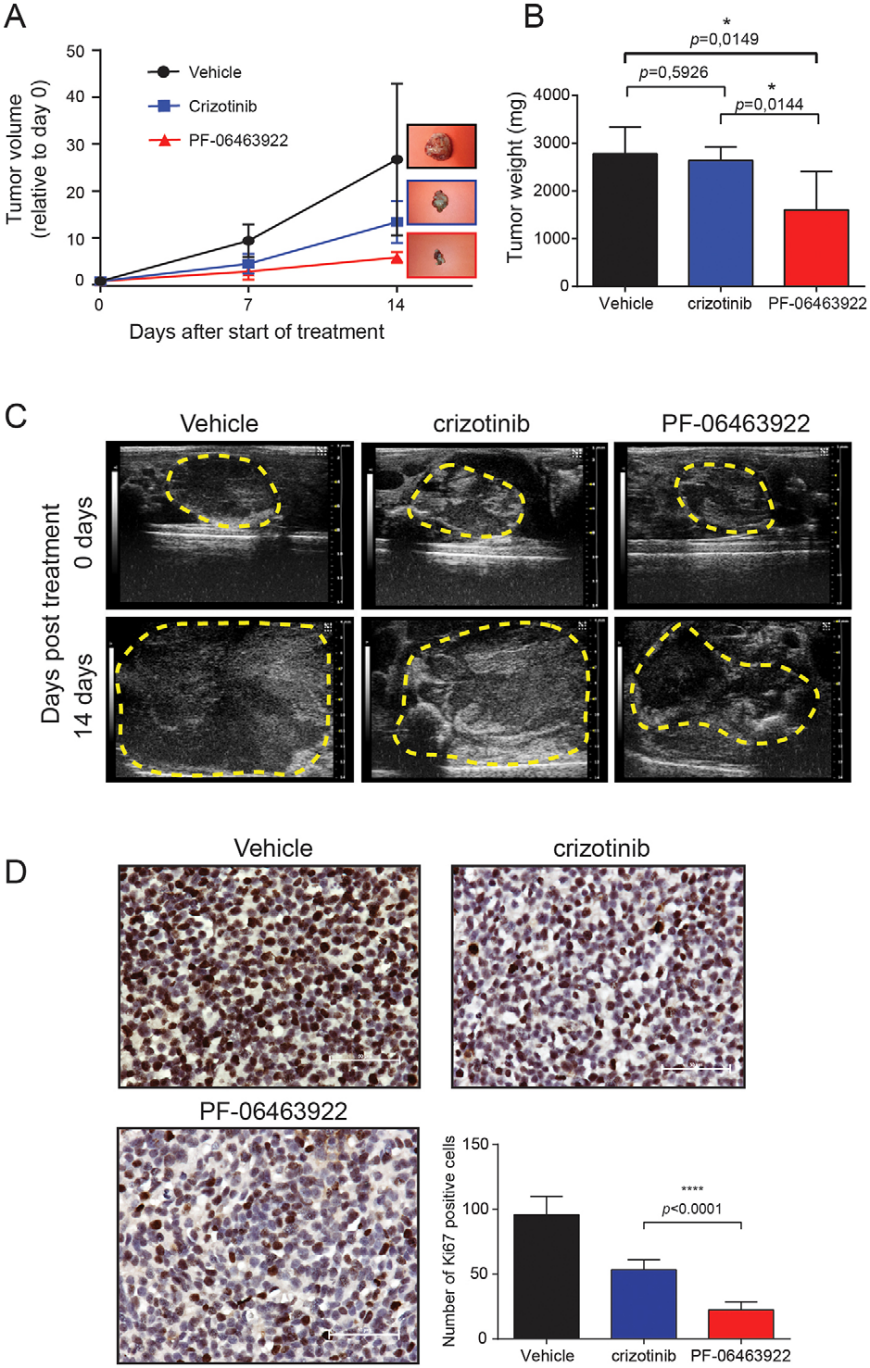
**Evaluation of preclinical efficacy of PF-06463922 in an orthotopic CLB-BAR xenograft neuroblastoma setting.** 2×10^5^ CLB-BAR cells were injected into the left adrenal gland of nude mice and adrenal tumor growth imaged by high-resolution ultrasonography using a Vevo 2100 Imaging system with MS-550D (22-55 MHz) linear array transducers (VisualSonics). (A) Tumor growth curves represented as relative tumor volume of vehicle, crizotinib or PF-06463922 treatment over 14 days compared with volume at day 0. Error bars show standard deviation between individual tumors (*n*=15 for each group). Statistical analysis indicates a significant difference between vehicle and PF-06463922 treatments at day 14, *P*<0.015 employing unpaired *t*-test. (B) Representative tumor weights in mg from mice treated with vehicle, crizotinib or PF-06463922. Statistical analysis shows significant difference between crizotinib and PF-06463922, and between vehicle and PF-06463922, *P*<0.05 using unpaired *t*-test. Data represented as mean±s.d. (C) Representative ultrasound images depicting tumor response to vehicle, crizotinib or PF-06463922 as single agent after 14 days of treatment. (D) Immunohistochemical staining of tumors for Ki-67 as a measure of proliferation rate as indicated. Ki67-positive cells were counted manually per field of vision and quantitative results presented as mean±s.d. (*n*=15).

To investigate the effectiveness of PF-06463922 *in vivo* we orthotopically injected human neuroblastoma cells (CLB-BAR, amplified *MYCN/ALK, ALKΔexon 4-11*) into the adrenal glands of BalbC/nude mice, prior to treatment with either crizotinib or PF-06463922. Treatment for 14 days with crizotinib (100 mg/kg) (Berry et al., 2012) inhibited the growth of orthotopically xenografted tumors in the adrenal glands with efficacy similar to that exhibited earlier with subcutaneously injected xenografts (Fig. 4A-C) (Umapathy et al., 2014). Treatment with PF-06463922 (10 mg/kg, once per day), resulted in a more profound decrease in tumor size (Fig. 4A-C). Excised tumor material from mice treated with either crizotinib or PF-06463922 exhibited reduced proliferation, with PF-06463922 exhibiting a greater decrease (Fig. 4D). Therefore, in orthotopic xenografts PF-06463922 displays more potent anti-tumor activity when compared with crizotinib.

**Fig. 5.**
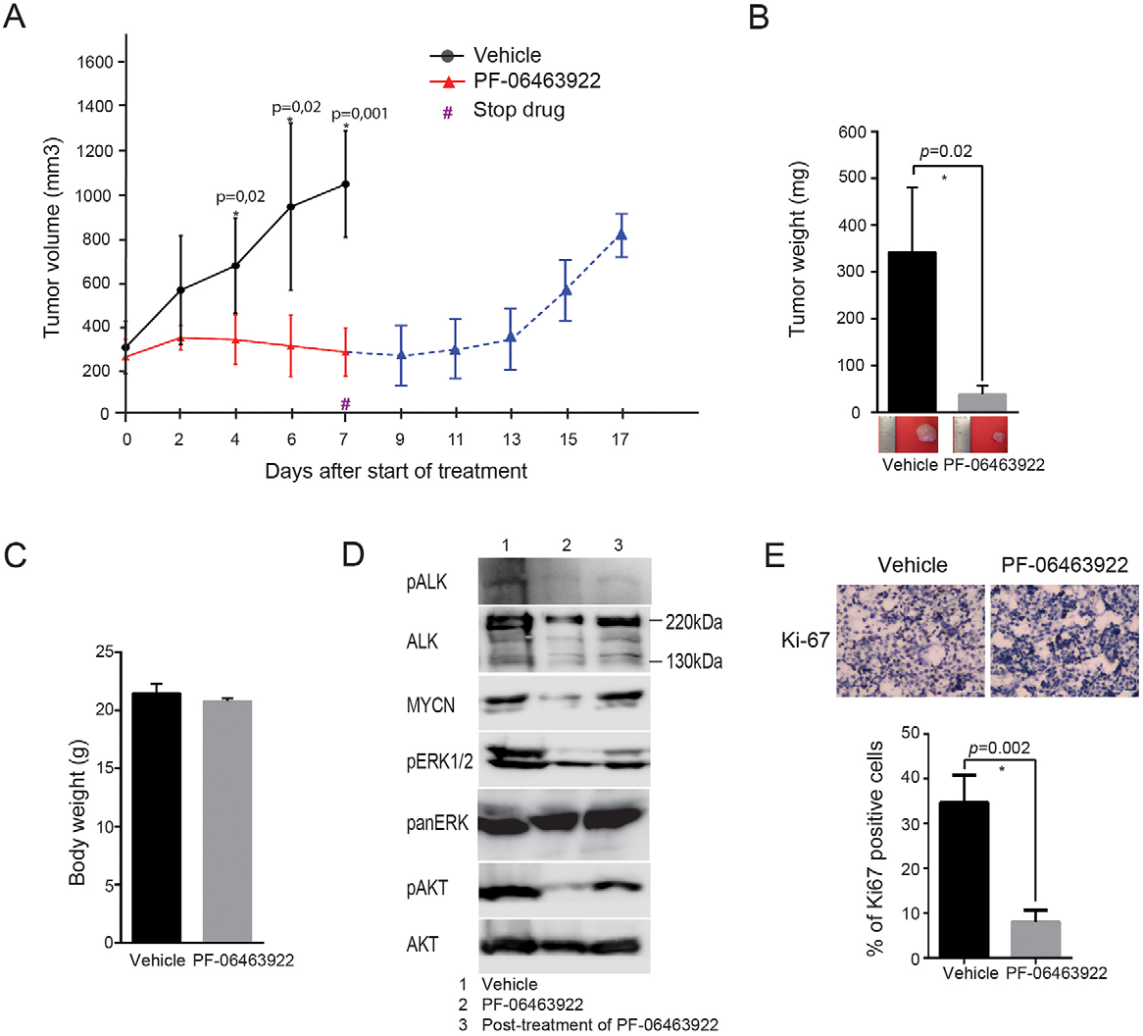
**Efficacy of PF-06463922 in an ALK^F1174^ CLB-GE xenograft neuroblastoma model.** 4.5×10^6^ CLB-GE cells were injected into left shoulder subcutaneously. (A) Tumor growth curves represent the average volume of vehicle group and PF-06463922-treated group, *P*≤0.05 (*n*=5 for each group). (B) Average tumor weights in vehicle group and PF-06463922 group are displayed, *P*=0.02. (C) Average body weight on the day of sacrifice is shown, *P*>0.05. (D) Immunoblotting analysis of indicated proteins from tumors collected after 8 days of treatment with vehicle or PF-0646399 and relapsed tumors. Tumor lysates were harvested, pre-cleared and analyzed by western blotting for ALK, phospho-ALK-Y1278, MYCN, phospho-ERK1/2 and phospho-AKT. Pan-ERK and pan-Akt were employed as loading controls. (E) Immunohistochemical staining of tumors for Ki-76 as a measure of proliferation rate as indicated. Ki67-positive cells were counted manually per field of vision and quantitative results (*n*=15). Statistical analysis shows significant difference between vehicle and PF-06463922-treated group, *P*<0.002 using unpaired *t*-test. Data in all graphs presented as mean±s.d.

We next investigated anti-tumor efficacy of PF-06463922 with neuroblastoma cells expressing ALK^F1174V^. For this we employed CLB-GE cells, injected subcutaneously in nude mice allowing tumor growth to be monitored on a daily basis. Animals with tumor growth were treated with PF-06463922 (10 mg/kg, twice per day, *n*=5) with dosing guided by previous reports (Yamazaki et al., 2014; Zou et al., 2015). In this model, PF-06463922 demonstrated significant anti-tumor activity with no increase of tumor size during treatment and no observed loss of body weight during treatment (Fig. 5A-C). After 7 days of treatment, three mice were euthanized and treatment was discontinued for the remaining animals. Tumor re-growth was observed 7 days after cessation of therapy (Fig. 5A). Inhibition of phosphorylated ALK, ERK1/2 and PKB/Akt was observed as well as reduced expression of MYCN in PF-06463922-treated tumors, however, the presence of MYCN was seen in tumors re-grown after withdrawal of the drug (Fig. 5D). Similar to the orthotopic xenograft model above, tumors from mice treated with PF-06463922 exhibited reduced staining of the proliferation marker Ki-67 (Fig. 5E). Thus, in subcutaneous xenografts, PF-06463922 displays a potent anti-tumor activity.

### Preclinical efficacy of PF-06463922 in a murine model of *Th-ALK^F1174L^*/*MYCN*-driven neuroblastoma

**Fig. 6.**
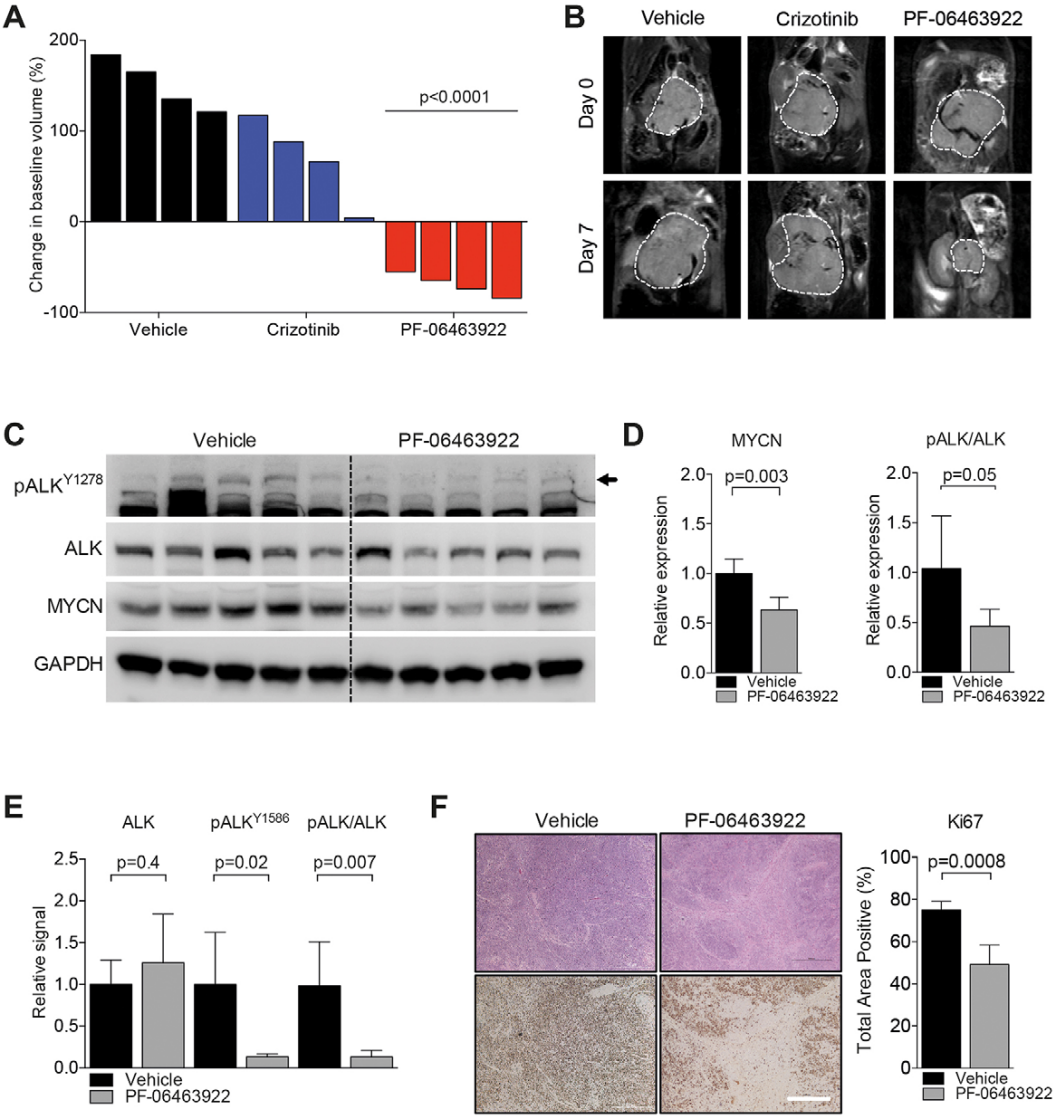
**Preclinical efficacy of PF-06463922 in a murine model of *Th-ALK^F1174L^/MYCN*-driven neuroblastoma.** (A) Waterfall plots of tumoral response in *Th-ALK^F1174L^/MYCN* mice treated with vehicle, crizotinib (100 mg/kg, once daily) or PF-06463922 (10 mg/kg, twice daily). Each bar indicates percent change in the volume of an individual tumor, as assessed by T_2_-weighted MRI, on day 7 compared with day 0 of treatment. (B) Representative MRI of each treatment group on day 0 and day 7 after treatment of indicated vehicle or drug. Hashed white line indicates tumor border. (C) Immunoblot analysis of phosphorylated ALK (phospho-ALK-Y1278), total ALK, and MYCN of tumors treated for 2 days with vehicle or PF-06463922. GAPDH was employed as loading control. Arrow indicates pALK-Y1278. (D) Quantification of band intensity of MYCN relative to GAPDH (left) and ratio of band intensity of pALK over total ALK (right) comparing vehicle versus treated samples. (E) Meso Scale Discovery assay depicted as electrochemiluminescence signal of treated samples relative to the respective vehicle controls for total ALK, phospho-ALK-Y1568, and the ratio of pALK to ALK. (F) Representative H&E (top) and immunohistochemical images for Ki67 (bottom) of vehicle and treated samples. Quantification of overall percentage of area positive for Ki67 (right). Error bars represent s.d. between individual animals (per group: *n*=4 in A, *n*=5 in C-F). *P*-values equal unpaired *t*-test comparison between vehicle and treatment groups.

We have previously shown that transgenic mice co-expressing an active ALK^F1174L^ mutation with MYCN via the tyrosine hydroxylase promoter potentiates neuroblastoma development characterized by earlier onset, higher penetrance and enhanced lethality (Berry et al., 2012). *Th-ALK^F1174L^/MYCN* mice respond with limited efficacy to single-agent treatment with crizotinib (Berry et al., 2012). Therefore, we examined the therapeutic effect of PF-06463922 as a single agent in the *Th-ALK^F1174L^/MYCN* model. Mice were treated for 7 days with vehicle, crizotinib (100 mg/kg) (Berry et al., 2012) or PF-06463922 (10 mg/kg) (Huang et al., 2014; Johnson et al., 2014; Yamazaki et al., 2015; Zou et al., 2015), and changes in tumor burden documented by serial MRI. Crizotinib treatment did not lead to a significant reduction in tumor volume (Fig. 6A,B), in agreement with earlier findings (Berry et al., 2012). In contrast, treatment with PF-06463922 for 7 days significantly reduced tumor growth rates, and tumor regression was displayed in these four mice. PF-06463922 was then administered in a short time course of 48 h in a second cohort of mice to assess pharmacodynamics and molecular changes resulting from drug treatment (Fig. 6C-F). In this cohort, reduced ALK Y1278 phosphorylation was found upon western blotting (Fig. 6C,D). Further, we developed a Meso Scale Discovery assay for higher sensitivity detection of phosphorylated ALK (E.R.T., L.S.D. and L.C., unpublished) in which we detected significantly lower signals of absolute levels of phosphorylated ALK Y1586, as well as the ratio of pALK to total ALK, in treated versus control samples (Fig. 6E). Additionally, we found a significant treatment effect of decreased levels of MYCN in PF-06463922-treated samples (Fig. 6C,D). Significantly, an immunohistochemical analysis of proliferation, assayed by Ki67 detection, demonstrated a high degree of intra-tumoral heterogeneity but an overall decrease in areas of proliferation (Fig. 6F). In agreement with our observations in the CLB-BAR and CLB-GE neuroblastoma cell lines, a trend of increased apoptosis was observed in the tumor material treated for 48 h although this was not significant (Fig. S1B). Thus, in addition to the potent inhibition seen in cell culture models and *in vitro* kinase assays, PF-06463922 exhibits robust activity towards tumor growth in a transgenic ALK-driven mouse model of neuroblastoma.

## DISCUSSION

The concept that ALK alteration can act as a bona fide oncogenic driver mutation in poor-outcome cancers of both adult and pediatric origin, and can be safely inhibited in a robust and selective manner using widely available drugs has generated significant interest within pediatric clinical oncology. ALK inhibitors are now well accepted as frontline agents with efficacy for ALK positive NSCLC (Awad and Shaw, 2014), although duration of response is tempered by the emergence of therapy resistant mutations (Choi et al., 2010; Doebele et al., 2012; Katayama et al., 2014, 2012). As in adult patients, excellent and in some cases complete responses have been reported in the pediatric literature, generally in those patients with ALK fusion positive tumors (Mosse et al., 2013). Available clinical data does not as yet support any conclusion about the development of therapy resistance in children on continuous treatment for ALK-fusion positive tumors, although case reports exist in the inflammatory myofibroblastic tumor (IMT) literature (Mosse et al., 2013).

In contrast to the strong response observed in ALK fusion-positive tumors, patient responses in ALK mutant neuroblastoma are less encouraging (Mosse et al., 2013). This relates either to clinical factors unique to the etiology of this form of neuroblastoma, or alternatively to other issues such as ligand binding or the binding affinity of particular ALK inhibitors for the kinase domain of ALK (Guan et al., 2015). Initial experience has shown it is difficult to inhibit growth of ALK-positive neuroblastoma with crizotinib in the clinic even at high dosing levels, motivating investigation of PF-06463922 with its higher affinity for ALK in this setting. Although ∼7% of neuroblastoma is ALK-positive at diagnosis (De Brouwer et al., 2010), recent data indicate that the incidence of ALK mutations may be 20-25% in patients with relapsed neuroblastoma, regardless of initial ALK status. This suggests that ALK may play a more important role in the development of neuroblastoma than previously thought (Schleiermacher et al., 2014).

The development of ALK-targeted therapies, such as crizotinib, has changed treatment of ALK-driven cancers, presently most visible in ALK-positive NSCLC. Given the accumulated basic knowledge concerning ALK mutants in neuroblastoma (Hallberg and Palmer, 2013 and references therein), and the potential that one of the newly developed ALK inhibitors could be effective for the neuroblastoma patient population, we aimed to explore next-generation ALK inhibitors in the preclinical setting, with a focus on PF-06463922 (lorlatinib). This cyclic small-molecule drug was recently described as a selective inhibitor of both ALK and ROS1, and has the ability to penetrate the central nervous system with a half-life of 2.7 h in a preclinical animal model (Johnson et al., 2014; Zou et al., 2015). Further, treatment of EML4-ALK-driven brain metastases with PF-06463922 led to regression of tumor volume and prolonged mouse survival, an effect that was reversible upon withdrawal of the inhibitor (Zou et al., 2015). PF-06463922 is currently in a phase 1 and 2 clinical trial (NCT01970865) for treatment of ALK-positive NSCLC. Although PF-06463922 has been shown to have increased activity compared with crizotinib on the EML4-ALK^L1196M^ gateway and other resistance mutations from NSCLC patients (Johnson et al., 2014; Zou et al., 2015), it has not been explored in neuroblastoma. The increased affinity of PF-06463922 for EML4-ALK^L1196M^ suggests that this ALK inhibitor may be more effective against the ALK activating mutations described in neuroblastoma. Of note, resistance mutations in the ALK fusion proteins are generally found in the vicinity of the ATP-binding site, whereas in neuroblastoma the majority of the mutations are in activation segments of the kinase domain, such as the αC helix and the activation loop (Hallberg and Palmer, 2013).

Neuroblastoma is a complex heterogeneous disease exhibiting a variety of characteristic patterns of genetic aberrations that correlate with clinical outcome, of which cases with ALK mutation and MYCN amplification are more aggressive in nature (De Brouwer et al., 2010). In the preclinical setting, crizotinib is not effective as a mono-drug treatment alternative in mouse models with co-expression of ALK and MYCN. Expression of both an active ALK^F1174L^ mutation together with MYCN overexpression in mice potentiates the development of neuroblastoma with earlier onset, higher penetrance and enhanced lethality (Berry et al., 2012), which upon treatment with crizotinib shows some degree of growth inhibition compared with vehicle treated mice (Berry et al., 2012). Similarly, single treatment with crizotinib in human neuroblastoma xenograft models expressing ALK­-addicted cell lines reduces tumor growth but does not substantially debulk or eradicate tumors (Umapathy et al., 2014). It is unclear whether this failure to achieve therapeutic benefit is related to the reduced affinity of crizotinib for the ATP-binding pocket of oncogenically activated ALK mutants, the overexpression of MYCN often observed in neuroblastoma, or a combination of these and other factors. Other important considerations are less well­-understood genetic mutations often observed in neuroblastoma patients, such as deletion of parts of chromosomes 1 and 11, or gain of chromosome 17q (Pugh et al., 2013).

An additional alternative hypothesis takes into account the locations of ALK mutations in therapy-induced resistance mutations of NSCLC and primary therapy-naïve mutations found in neuroblastoma. Secondary mutations in ALK that emerge on patient treatment with ALK inhibitors in the clinic, e.g. for NSCLC, generally either cluster around the inhibitor/ATP-binding site and effect inhibitor binding and/or are more distal and increase ALK catalytic activity. A few resistance mutations overlap with neuroblastoma ALK mutations from familial and somatic cases that are located at or near residues important for kinase activation, such as the activation loop and the αC helix (Hallberg and Palmer, 2013). In our analyses, two secondary NSCLC mutations, ALK^G1269A^ and ALK^F1174L^, display intrinsic activation of full-length ALK in the absence of external stimuli. Furthermore, we observe differences between PF-06463922 and crizotinib treatment in both the Ba/F3 transformation assay and the *in vitro* kinase assay model systems. With crizotinib, the ALK^F1174L^ mutation exhibits a ∼twofold higher IC_50_ when compared with wild-type ALK in both models. In contrast, PF-06463922 treatment inhibits kinase activity of the ALK^F1174L^ mutant within a similar range to that of wild-type ALK. Importantly, PF-06463922 abrogates ALK activity in the single-digit nanomolar range in all our different preclinical assays, including the neuroblastoma hot-spot mutations, ALK^F1174^, ALK^F1245^ and ALK^R1275^ (Bresler et al., 2014; De Brouwer et al., 2010). These results suggest that PF-06463922 is a superior therapeutic option for neuroblastoma patients carrying ALK mutations.

Patients with ALK-positive neuroblastoma lack any suitable options for current treatment, despite expression of a bona fide oncogene that has been successfully targeted with crizotinib in other pediatric malignancies. The preclinical data presented here clearly show that PF-06463922 is more effective than crizotinib in the inhibition of ALK mutants found in neuroblastoma patients. All neuroblastoma mutations tested respond to PF-06463922 inhibition. The cell line, *in vitro* kinase and Ba/F3 cell assays presented here are strongly consistent with rapid and robust abrogation of tumor growth in both human implanted and murine endogenous tumors driven by highly resistant ALK mutations. Together, these results suggest that PF-06463922 should be further explored as a therapeutic agent in ALK-positive pediatric neuroblastoma.

## MATERIALS AND METHODS

### Reagents

Primary antibodies were: phospho-ALK (Y1604 (1:2000; 3341) and Y1278 (1:2000; 12127), phospho-ERK1/2, actin (1:2000; 4967), phospho-Akt (1:2000; 92711), MYCN (1:2000; 9405), Ki67 (1:100; 9129), GADPH and phospho-ERK5 (1:1000; 3371) from Cell Signaling Technology (Danvers, MA). Pan-ERK1/2 (1:2000; 610123), was purchased from BD Transduction Laboratories (Franklin Lakes, NJ). Ki67 was from BD Pharmingen, (1:100; 558616). Monoclonal antibody 135 (anti-ALK) was described (Chand et al., 2013; Moog-Lutz et al., 2005). Horseradish-peroxidase-conjugated secondary antibodies goat anti-rabbit IgG (PIEA31460) and goat anti-mouse IgG (PIEA31394; both at 1:5000) were from Thermo Scientific (Waltham, MA).

Crizotinib was from Haoyuan Chemexpress Co., Limited, Shanghai, China. PF-04643922 was from Pfizer, California, USA.

### CLB-BAR, CLB-GE, CLB-PE, SK-N-AS and IMR32 neuroblastoma cell analysis

The neuroblastoma CLB cell lines were from Valerie Combaret (Centre Leon Berard, France) under material transfer agreement and all cell lines were authenticated during the investigation with Affymetrix Cytoscan High Density array and compared with earlier cytoscans (Schleiermacher et al., 2003). CLB-BAR, CLB-GE, CLB-PE, SK-N-AS and IMR32 neuroblastoma cells (0.25×10^6^) were plated on collagen-coated 48-well plates. Cells were treated with increasing concentrations of inhibitors as indicated and followed for 5 days as described (Chand et al., 2013). Immunoblotting analysis of inhibition of ALK signaling was performed with indicated antibodies as described (Chand et al., 2013).

### Ba/F3 assay, PC12 culture assay and IC_50_ determination

Ba/F3 cells were grown as described (Chand et al., 2013). Cell viability was tested with resazurin (Sigma, Stockholm, Sweden) (O'Brien et al., 2000). IC_50_ values were determined for individual cell lines and experiments were carried out at least three times independently in triplicates.

### PC12 cell culture, assay and IC50 determination

PC12 cells were grown and transfected with various ALK constructs as described (Chand et al., 2013). 48 h after transfection, cells were treated with different concentrations of crizotinib (from 0 to 5000 nM) or PF-06463922 (from 0 to 500 nM) for 2 h. Cells were then washed twice with PBS and harvested as described (Schonherr et al., 2012, 2011). Samples were boiled in SDS sample buffer and analyzed by immunoblotting. Intensity of pALK (Y1604) and total ALK bands was quantified with Image Studio Lite 3.1 (LI-COR). Data were normalized to the 0 nM inhibitor samples. IC_50_ values were calculated by fitting data to a log (inhibitor) versus normalized response (variable slope) equation in GraphPad Prism 6.0.

### Generation of recombinant ALK TKD proteins

DNA encoding ALK residues 1090-1416, harboring the entire tyrosine kinase domain, was amplified with Phusion High-Fidelity DNA polymerase (ThermoScientific) and cloned into pFastBac/NT-TOPO vector (Invitrogen) for expression of 6× histidine-tagged recombinant ALK TKD proteins in *Spodoptera frugiperda* Sf21 cells. Recombinant baculovirus was generated using the Bac-to-Bac baculovirus expression system (Invitrogen). Sf21 cells were infected with P2 virus stock for 3 days at 27°C prior to lysis in native Ni-NTA lysis buffer (50 mM NaH_2_PO_4_ pH 8.0, 300 mM NaCl, 10 mM imidazole, 1% Triton X-100 and protease inhibitor cocktail). His-tagged protein was recovered from the lysis supernatant using Ni-NTA His•Bind resins (Novagen). After two washes, resins were incubated in 50 mM Tris-Cl pH 7.4, 150 mM NaCl, 10 mM MgCl_2_ and 2 mM ATP at 30°C for 1 h to achieve full ALK TKD autophosphorylation. After two additional washes, ALK TKD proteins were eluted and their concentrations were determined by absorbance at 280 nm. Purity of ALK TKD proteins was assessed by SDS-PAGE and Coomassie Blue staining.

### *In vitro* kinase assays

Assays were performed employing a peptide mimic of the ALK activation loop with sequence ARDIYRASYYRKGGCAMLPVK, referred to as YYY peptide (Donella-Deana et al., 2005) and using radiolabeled ATP as described previously (Hastie et al., 2006). IC_50_ values were calculated by fitting data to a log (inhibitor) versus normalized response (variable slope) equation in GraphPad Prism 6.0. All data are shown as mean±s.d. from at least two independent experiments.

### Subcutaneous and orthotopic xenografts

Balbc/nude mice (Taconic) at 6 to 8 weeks of age were orthotopically injected with neuroblastoma cells (2×10^5^ CLB-BAR cells) in the left adrenal gland via a left subcostal incision under 2% isoflurane anesthesia using a 22 gauge needle attached to a Hamilton syringe. Orthotopic tumor growth was monitored after 10 days of injection and mice bearing 30 mm^3^ to 60 mm^3^ established tumors were equally treated with either vehicle, 100 mg/kg crizotinib or 10 mg/kg PF-06463922 once per day (oral gavage) administrated daily for 14 days of treatment. MRI measurement of tumor volume of orthotopic xenograft was calculated as described (Umapathy et al., 2014).

Balbc/nude mice (Janvier Lab, France) at 5-6 weeks age were subcutaneously injected with neuroblastoma cells, (4.5×10^6^ CLB-GE cells) mixed with 50 µl matrigel to the left front shoulder. Once tumor volume reached 300 mm^3^, drug was given at 10 mg/kg body weight, by oral gavage, twice daily, continuously for 7 days. Mice were grouped by tumor volume randomly. Tumor volume was calculated by the following equation: V=0.5236×L×W^2^ (V=volume, L=longest, W=width). PF-06463922 and crizotinib were formulated in 90% polyethylene glycol and 10% 1-methyl-2-pyrrolidinone solution. Immunohistochemistry was as described (Yamazaki et al., 2014). After mounting and drying, images were taken using light microscopy (Leica). Ki67-positive cells were counted manually per field of vision and quantitative results presented as mean±s.e.m. (*n*=15). Statistical analysis between crizotinib and PF-06463922 samples was performed by *t*-test.

### Murine models and therapeutic trials

*Th-MYCN* hemizygous mice (Weiss et al., 1997) were bred with *Th-ALK^F1174L^* hemizygous mice (Berry et al., 2012). Animals hemizygous for both transgenes were palpated for intra-abdominal tumors twice weekly. Animals with palpable tumors (30-50 days old) were randomized to treatment groups. For seven-day intervention trials, mice were treated with 10 mg/kg PF-06463922 (twice daily via oral gavage), 100 mg/kg crizotinib (once daily via oral gavage), or vehicle. For two-day intervention trials, mice were treated with PF-06463922 5 mg/kg (twice daily via oral gavage), or vehicle. Volumetric MRI and immunoblotting assays were performed as described (Berry et al., 2012; Jamin et al., 2013). MSD 96-well assays were carried out as per the manufacturer's protocol with development of the assay to detect total ALK and phosphorylated ALK (E.R.T., L.S.D., and L.C., unpublished). Briefly, plates were coated with anti-ALK antibody (3791 from Cell Signaling Technology, Danvers, MA) overnight, followed by a series of blocking (TBS +0.1% Tween-20 +5% BSA) and washing (TBS +0.1% Tween-20) before incubation overnight with protein from tumor lysates. This was followed by incubation with primary antibodies for detection of total ALK (1:2000; 3633) and pALK Y1586 (1:2000; 3348) (both from Cell Signaling Technology, Danvers, MA) subsequent to incubation with a secondary antibody (1 µg/ml; SULFO-tag, R32AB-1, Meso Scale Diagnostics) for assay readout with a SECTORTM 6000 instrument. Ruthenium counts for phosphorylated ALK and total ALK were corrected for background by subtracting signal emitted from wells containing lysis buffer alone.

Mouse tissue samples were fixed in 10% neutral-buffered formalin, processed, and embedded in paraffin, according to standard protocols. Sections (5 μm thick) were prepared for standard antibody detection and hematoxylin and eosine staining. Immunohistochemistry was conducted following the avidin-biotin immunoperoxidase staining procedure using anti-Ki67 antibody (1:100; clone B56, BD Pharmingen). Vector Imm PACT DAB (SK-4105) was used for detection, along with hematoxylin for counterstaining nuclei.

Statistical significance was determined using GraphPad Prism 6.0 software. Unpaired *t*-tests were used to compare data between groups. Statistical significance was defined as *P*<0.05. Error bars correspond to standard deviation between individual animals (*n*=4-5 for each group).

### Ethical permission

All *in vivo* experiments were with the permission of ethical committees in Umeå (A51-13) and Göteborg (230-2014), or in accordance with the local ethical review panel, and the UK Home Office Animals (Scientific Procedures) Act 1986 under PPL 70/7945.

## Note added in proof

During the review process a study was published (Infarinato et al., 2016) that supports our finding regarding PF-06463922 being a potent inhibitor of crizotinib-resistant patient and cell line xenografts.
